# Supplementary material for: Symmetry Breaking of Electronic Structure upon the π→π* Excitation in Anthranilic Acid Homodimer
Source: Molecules. 2024 Nov 25;29(23):5562. doi: 10.3390/molecules29235562 (PMC11643959; doi:10.3390/molecules29235562)
Supplement: Supplementary file 1 [file molecules-29-05562-s001.zip › molecules-3316022-supplementary.pdf]

Symmetry Breaking of Electronic Structure upon the π→π* Excitation in Anthranilic Acid Homodimer

Marcin Andrzejak [1,*], Joanna Zams [1,2], Jakub Goclon [3] and Przemysław Kolek [4,*]

[1] K. Gumiński Department of Theoretical Chemistry, Faculty of Chemistry, Jagiellonian University, 31-007 Kraków, Poland

[2] Doctoral School of Exact and Natural Sciences, Jagiellonian University, 31-007 Kraków, Poland; joanna.zams@doctoral.uj.edu.pl

[3] Faculty of Chemistry, University of Bialystok, 15-328 Białystok, Poland; j.goclon@uwb.edu.pl

[4] Institute of Physics, University of Rzeszów, 35-310 Rzeszów, Poland

* Correspondence: m.andrzejak@uj.edu.pl (M.A.); pkolek@ur.edu.pl (P.K.)

Supplementary Material

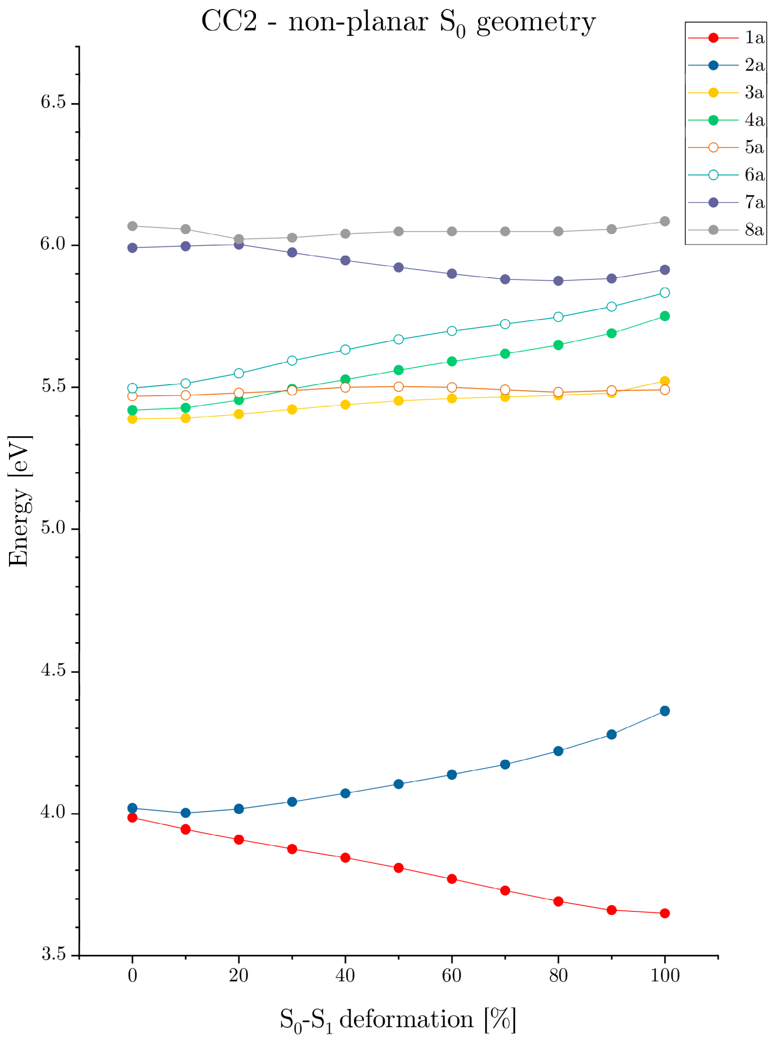

Figure S1. Landscape of excited state energies calculated for the lowest 8 states at the CC2 level of theory for a series of intermediate geometries between the non-planar ground state (C_i) structure and the structure optimized for the S_1 state (with no assumed planarity of the molecule, C_1 symmetry group).

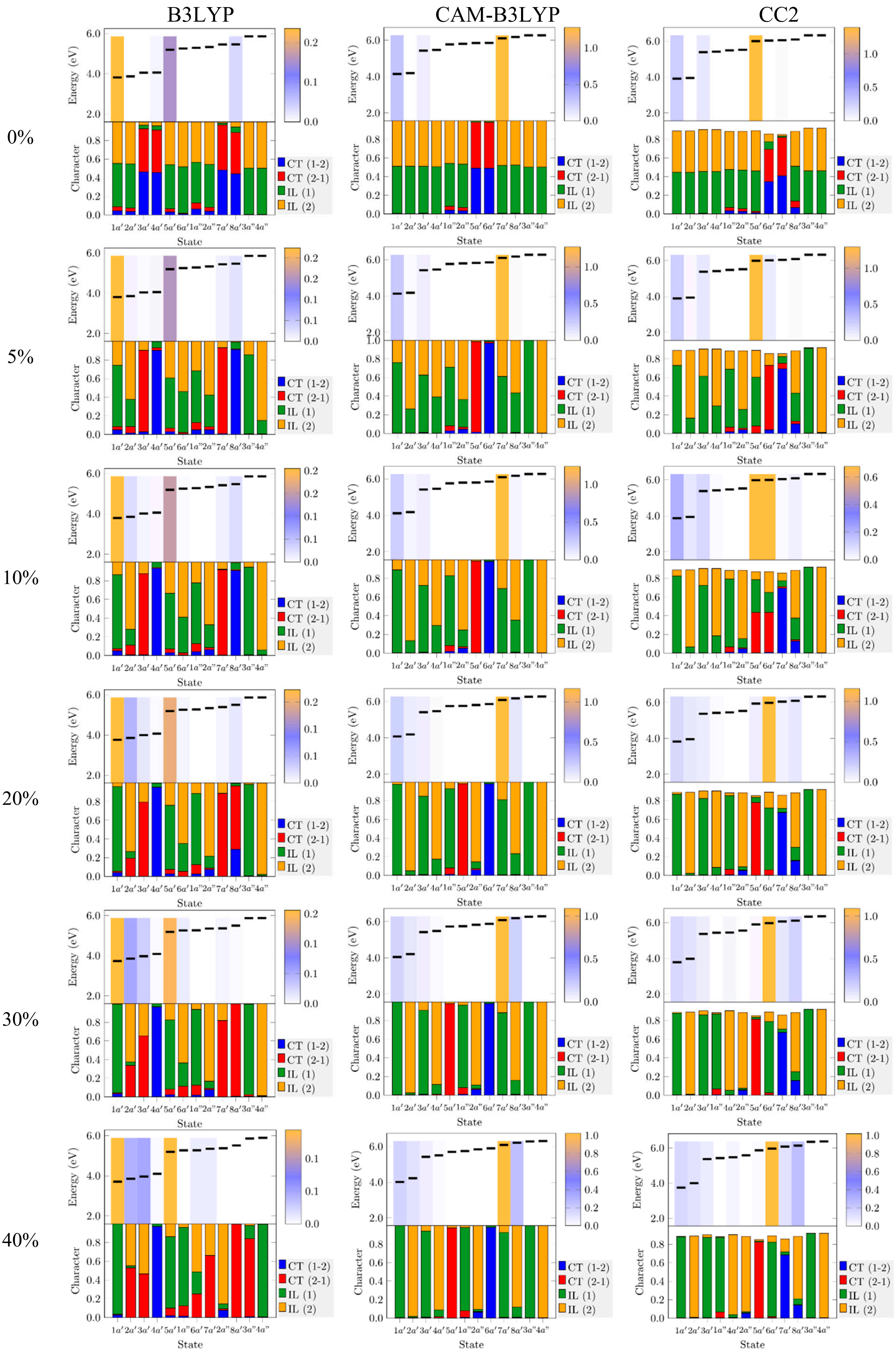
B3LYP
CAM-B3LYP
CC2
0%
5%
10%
20%
30%
40%
Energy (eV)
Character
State
CT (1-2)
CT (2-1)
IL (1)
IL (2)

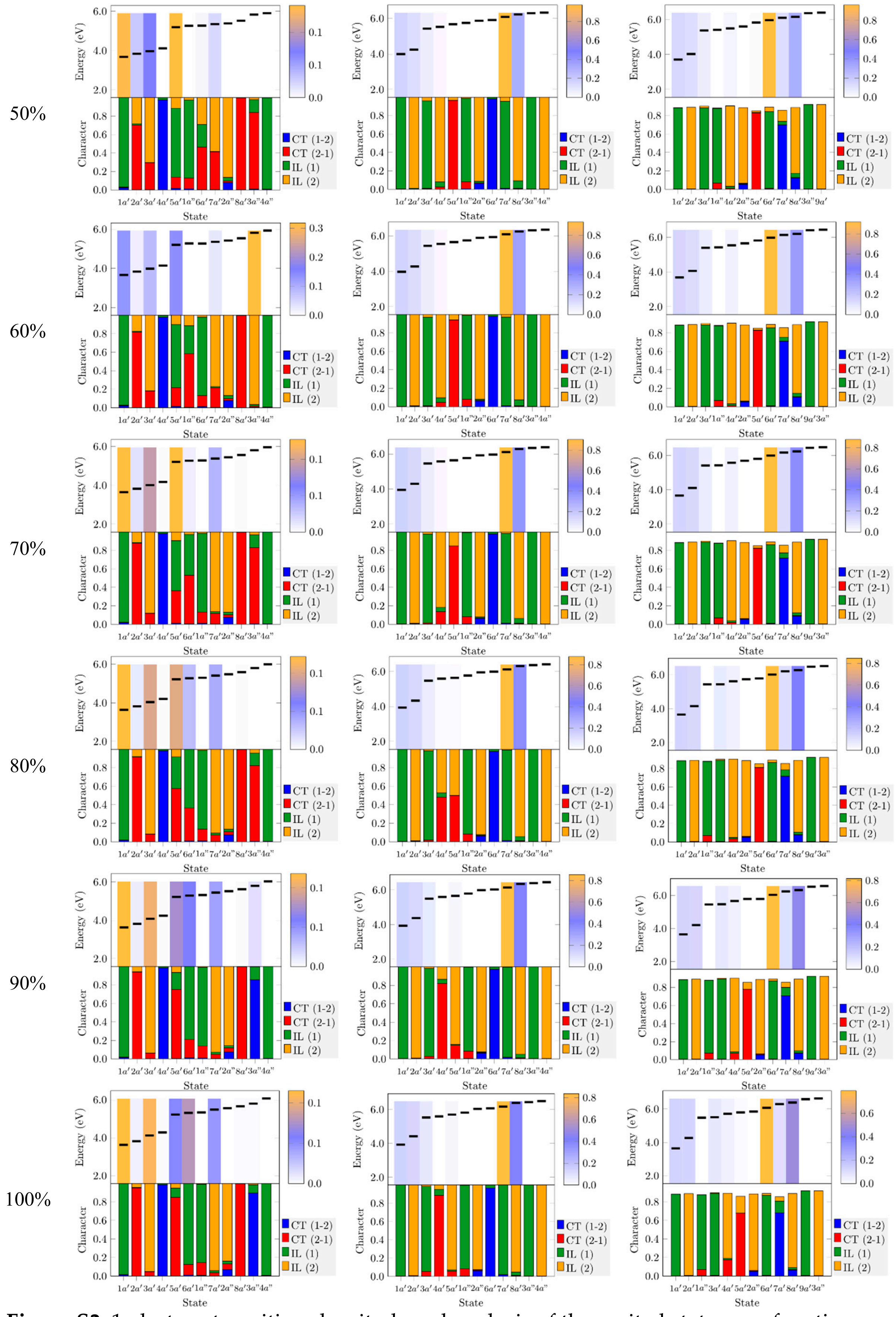

Figure S2. 1-electron transition-density-based analysis of the excited state wavefunctions, calculated for the lowest 12 states at different levels of theory for a series of intermediate geometries between the ground state (C_{2h}) structure and the structure optimized for the S_1 state (with the assumed planarity of the molecule).

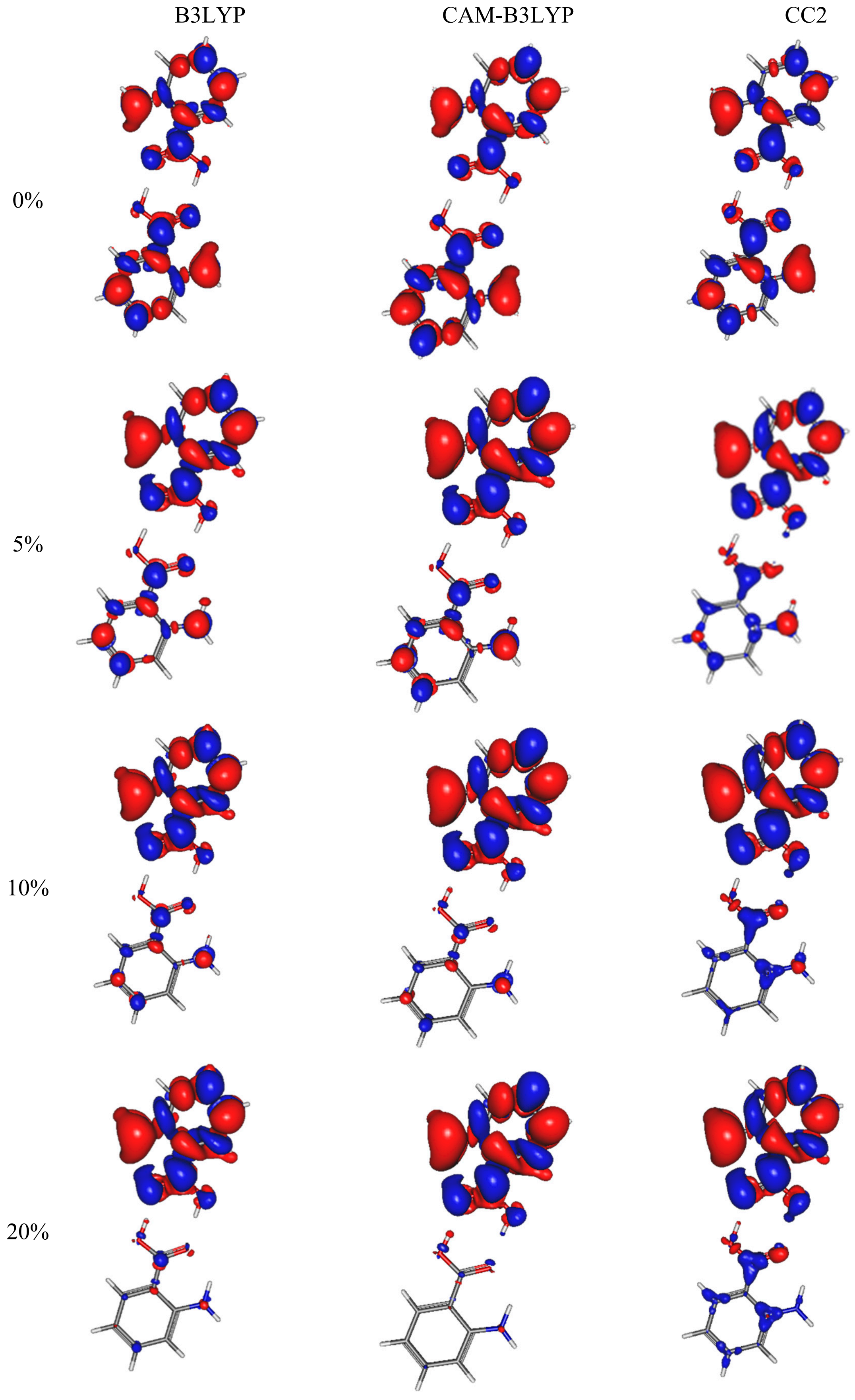
B3LYP
CAM-B3LYP
CC2
0%
5%
10%
20%

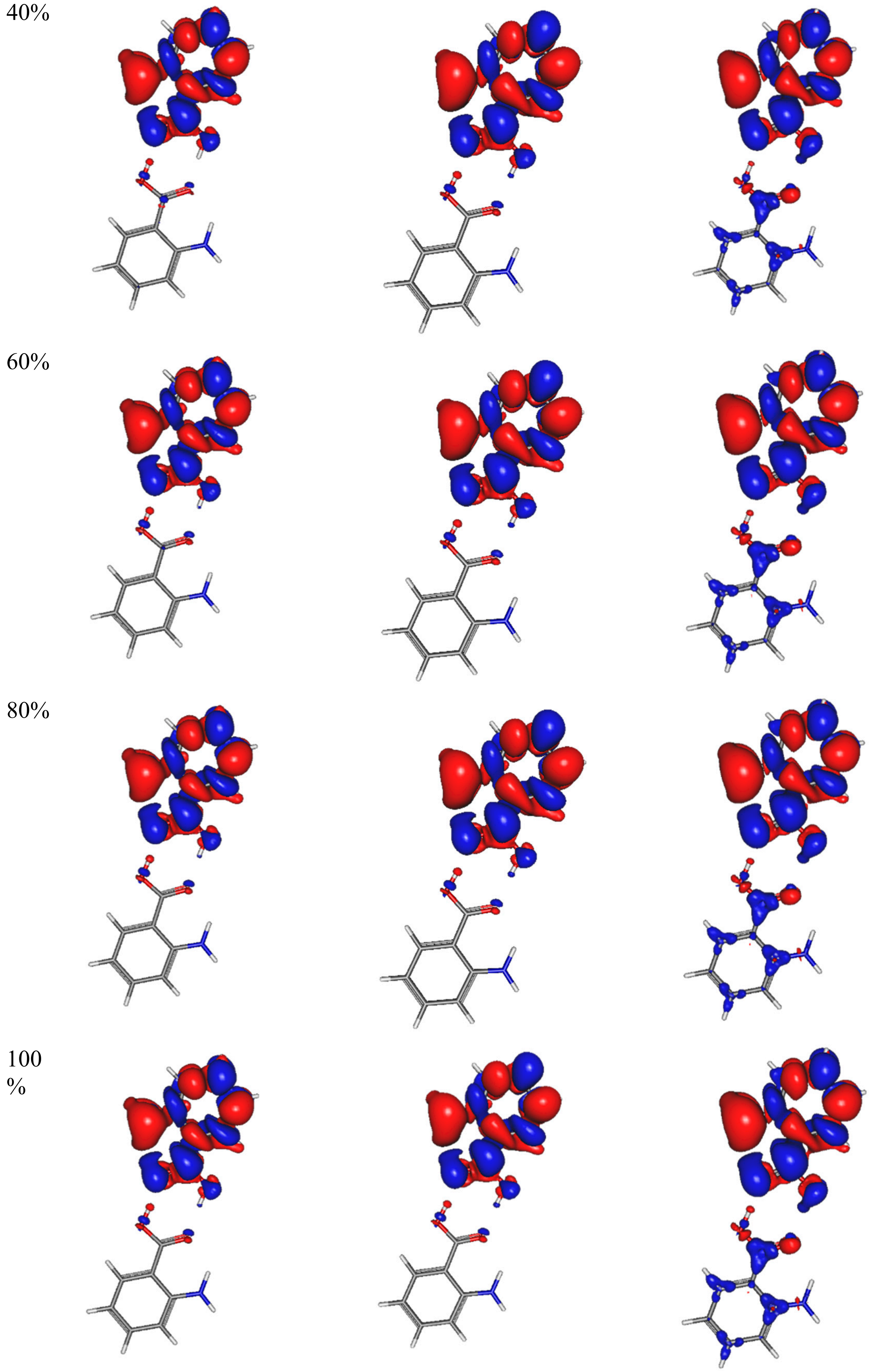

Figure S3. Difference densities for the S_0-S_1 transition, calculated at different levels of theory for a series of intermediate geometries between the ground state (C_{2h}) structure and the structure optimized for the S_1 state (with the assumed planarity of the molecule).

$S_0 \rightarrow S_1$ Natural Transition Orbitals - 0% distortion

No.	Occupied	Virtual
1 (50.31%)	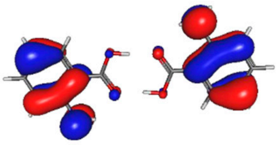	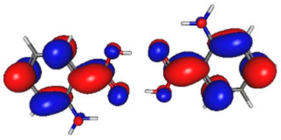
2 (43.75%)	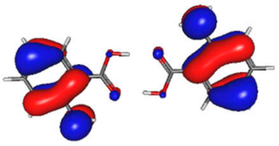	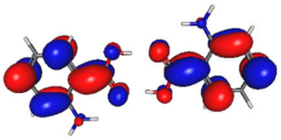
3 (2.52%)	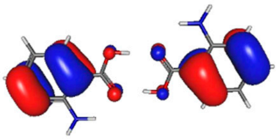	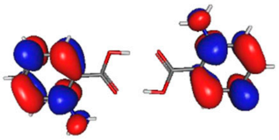
4 (2.49%)	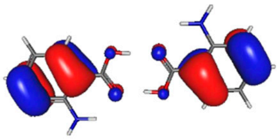	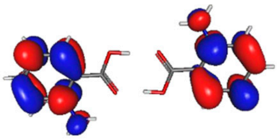
5 (0.09%)	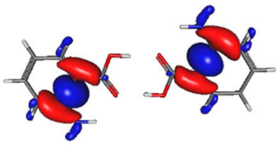	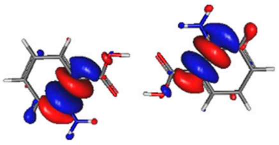
6 (0.09%)	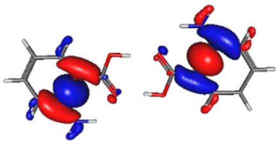	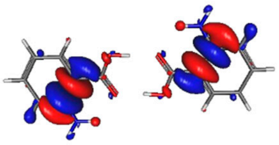

$S_0 \rightarrow S_1$ Natural Transition Orbitals - 5% distortion

No.	Occupied	Virtual
1<br>(77.07%)	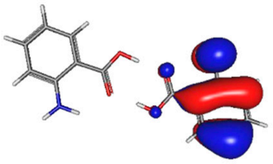	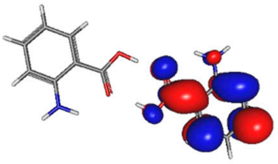
2<br>(17.02%)	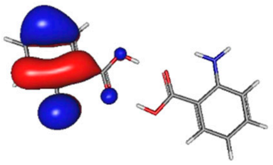	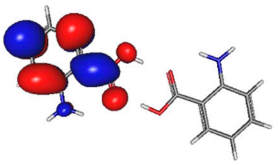
3<br>(4.13%)	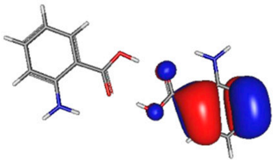	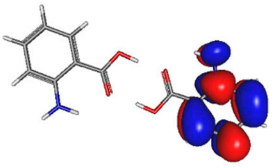
4<br>(0.85%)	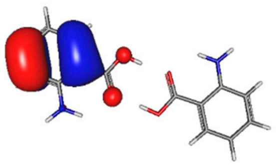	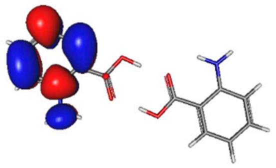
5<br>(0.15%)	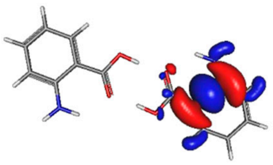	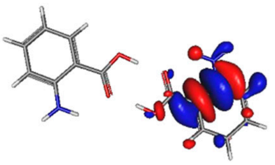
6<br>(0.12%)	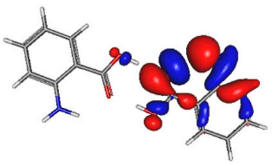	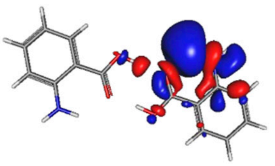

$S_0 \rightarrow S_1$ Natural Transition Orbitals - 10% distortion

No.	Occupied	Virtual
1 (87.32%)	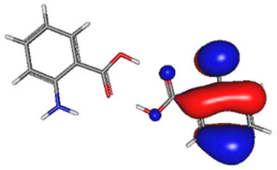	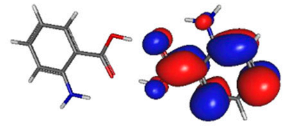
2 (6.82%)	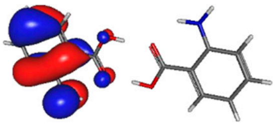	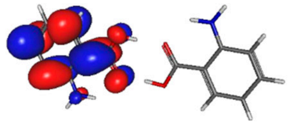
3 (4.62%)	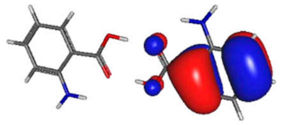	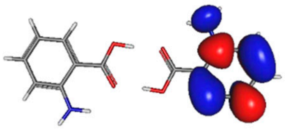
4 (0.31%)	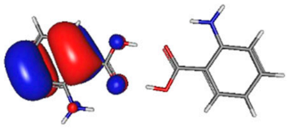	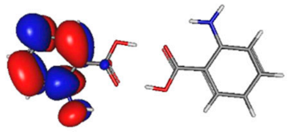
5 (0.18%)	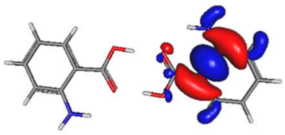	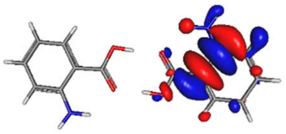
6 (0.14%)	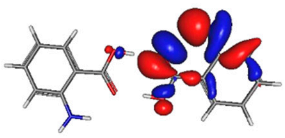	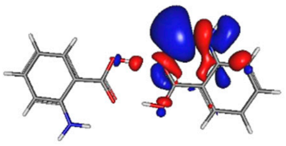

$S_0 \rightarrow S_1$ Natural Transition Orbitals - 20% distortion

No.	Occupied	Virtual
1 (92.25%)	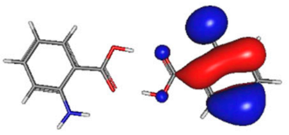	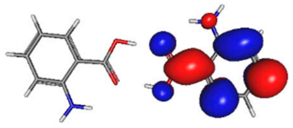
2 (4.67%)	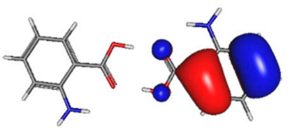	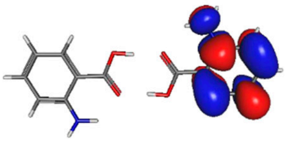
3 (2.06%)	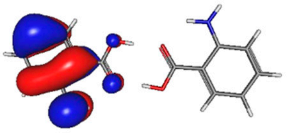	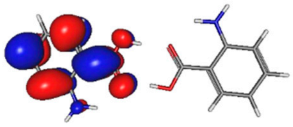
4 (0.19%)	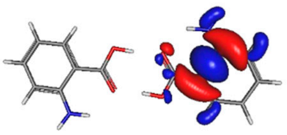	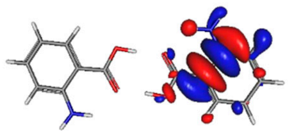
5 (0.15%)	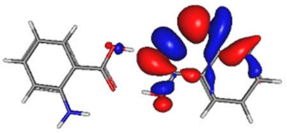	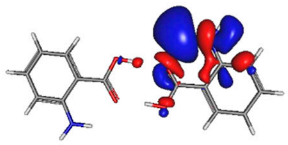
6 (0.12%)	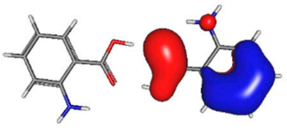	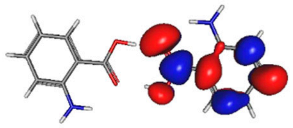

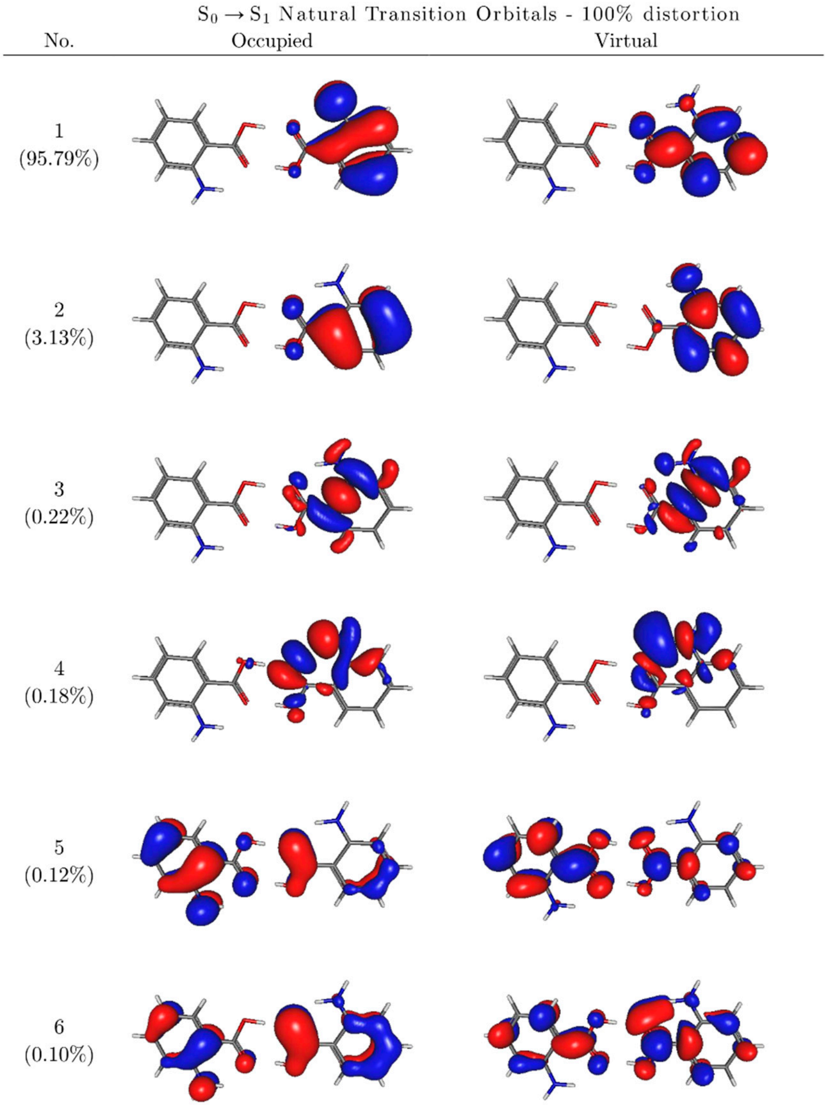

Figure S4. Natural transition orbitals for the S_0-S_1 transition calculated at the CC2/def2-TZVPP level of theory for the C_{2h} (ground state) structure, the S_1 state geometry, and several intermediate structures.

Table S1. Cartesian coordinates of the dimer used in our study obtained for the AA dimer at different levels of theory. All calculations used the Def2-TZVPP basis set.

1. CC2, planar, S_0 (C_{2h} symmetry group):

C	4.3081924	-0.1686043	0.0000000
C	3.2224273	-1.0849005	0.0000000
C	3.4782322	-2.4689375	0.0000000
C	4.7702141	-2.9657674	0.0000000
C	5.8423586	-2.0628899	0.0000000
C	5.6166331	-0.6974014	0.0000000
C	1.8370374	-0.6119957	0.0000000

H	2.6316537	-3.1401554	0.0000000
H	4.9472188	-4.0317791	0.0000000
H	6.8611128	-2.4288658	0.0000000
H	6.4533207	-0.0087971	0.0000000
N	4.1283865	1.1794739	0.0000000
H	4.9260315	1.7867936	0.0000000
H	3.1896748	1.5439400	0.0000000
O	1.5261265	0.6008781	0.0000000
O	0.9202540	-1.5724307	0.0000000
H	0.0104917	-1.1488401	0.0000000
C	-4.3081924	0.1686043	0.0000000
C	-3.2224273	1.0849005	0.0000000
C	-3.4782322	2.4689375	0.0000000
C	-4.7702141	2.9657674	0.0000000
C	-5.8423586	2.0628899	0.0000000
C	-5.6166331	0.6974014	0.0000000
C	-1.8370374	0.6119957	0.0000000
H	-2.6316537	3.1401554	0.0000000
H	-4.9472188	4.0317791	0.0000000
H	-6.8611128	2.4288658	0.0000000
H	-6.4533207	0.0087971	0.0000000
N	-4.1283865	-1.1794739	0.0000000
H	-4.9260315	-1.7867936	0.0000000
H	-3.1896748	-1.5439400	0.0000000
O	-1.5261265	-0.6008781	0.0000000
O	-0.9202540	1.5724307	0.0000000
H	-0.0104917	1.1488401	0.0000000

2. CC2 non-planar, S_0 (C_i symmetry group):

C	4.1998101	0.9828148	-0.0222153
C	3.3955887	-0.1862223	-0.0302663
C	4.0072222	-1.4536198	-0.0187113
C	5.3848589	-1.5904829	0.0076304
C	6.1800308	-0.4372451	0.0259691
C	5.5994466	0.8199007	0.0084720
C	1.9320130	-0.0991520	-0.0211328
H	3.3689376	-2.3252012	-0.0252559
H	5.8370285	-2.5719563	0.0185860
H	7.2589460	-0.5208306	0.0511537
H	6.2231078	1.7060348	0.0122439
N	3.6717074	2.2458837	-0.1019489
H	4.2677402	3.0002127	0.1932901
H	2.6851563	2.3253002	0.0965090
O	1.3100608	0.9844026	0.0436524
O	1.3061945	-1.2681678	-0.0801627
H	0.3160916	-1.1047121	-0.0594455
C	-4.1998101	-0.9828148	0.0222153
C	-3.3955887	0.1862223	0.0302663
C	-4.0072222	1.4536198	0.0187113
C	-5.3848589	1.5904829	-0.0076304
C	-6.1800308	0.4372451	-0.0259691
C	-5.5994466	-0.8199007	-0.0084720
C	-1.9320130	0.0991520	0.0211328
H	-3.3689376	2.3252012	0.0252559
H	-5.8370285	2.5719563	-0.0185860
H	-7.2589460	0.5208306	-0.0511537

H	-6.2231078	-1.7060348	-0.0122439
N	-3.6717074	-2.2458837	0.1019489
H	-4.2677402	-3.0002127	-0.1932901
H	-2.6851563	-2.3253002	-0.0965090
O	-1.3100608	-0.9844026	-0.0436524
O	-1.3061945	1.2681678	0.0801627
H	-0.3160916	1.1047121	0.0594455

3. CC2 planar, S_1 (C_s symmetry group):

C	4.2935697	-0.5658798	0.0000000
C	3.0853390	-1.3838328	0.0000000
C	3.2932085	-2.7720721	0.0000000
C	4.6114079	-3.3254575	0.0000000
C	5.7541588	-2.5326570	0.0000000
C	5.5749956	-1.1161016	0.0000000
C	1.7747324	-0.8017838	0.0000000
H	2.4357528	-3.4290388	0.0000000
H	4.7078093	-4.4037597	0.0000000
H	6.7432052	-2.9651175	0.0000000
H	6.4277139	-0.4470314	0.0000000
N	4.0886397	0.7728398	0.0000000
H	4.8682591	1.4146396	0.0000000
H	3.0842823	1.0585747	0.0000000
O	1.5369062	0.4673872	0.0000000
O	0.7675662	-1.6924864	0.0000000
H	-0.0951808	-1.1913637	0.0000000
C	-4.2917624	0.5565304	0.0000000
C	-3.1185691	1.3574624	0.0000000
C	-3.2338297	2.7591493	0.0000000
C	-4.4694351	3.3851764	0.0000000
C	-5.6273000	2.5961339	0.0000000
C	-5.5401685	1.2143979	0.0000000
C	-1.7849126	0.7451541	0.0000000
H	-2.3230007	3.3402967	0.0000000
H	-4.5372453	4.4637750	0.0000000
H	-6.6039373	3.0631747	0.0000000
H	-6.4420902	0.6135673	0.0000000
N	-4.2470160	-0.8032019	0.0000000
H	-5.1006750	-1.3286504	0.0000000
H	-3.3467453	-1.2557479	0.0000000
O	-1.6042582	-0.4933204	0.0000000
O	-0.7782742	1.6062623	0.0000000
H	0.0968539	1.0929811	0.0000000

4. CC2 non-planar, S_1 (C_1 symmetry group):

C	4.2111573	0.9678919	-0.0843180
C	3.3673486	-0.2203042	-0.0122539
C	4.0468040	-1.4369388	0.1544588
C	5.4731199	-1.4850972	0.2417712
C	6.2647589	-0.3442820	0.1650258
C	5.6019337	0.9094235	-0.0002510
C	1.9387567	-0.1431867	-0.1090981
H	3.4745621	-2.3510416	0.2161492
H	5.9401530	-2.4532680	0.3702468
H	7.3408997	-0.3962755	0.2316859
H	6.1652622	1.8335764	-0.0586039

N	3.5505964	2.1419606	-0.2239667
H	4.0557248	3.0138403	-0.2905807
H	2.5126038	2.0512039	-0.2913320
O	1.2747217	0.9491320	-0.2944352
O	1.3053476	-1.3243181	0.0011864
H	0.3258637	-1.1653407	-0.1004713
C	-4.2204031	-0.9703776	-0.1161189
C	-3.3890438	0.1763610	-0.0372629
C	-3.9687607	1.4440016	0.1492059
C	-5.3400876	1.6026144	0.2679442
C	-6.1610031	0.4699419	0.2019114
C	-5.6119704	-0.7874698	0.0108275
C	-1.9254348	0.0616467	-0.1138168
H	-3.3097980	2.2983946	0.2038939
H	-5.7667171	2.5849346	0.4136695
H	-7.2347850	0.5694445	0.2962980
H	-6.2555868	-1.6571769	-0.0508263
N	-3.7261220	-2.2255813	-0.3686018
H	-4.3183518	-2.9976834	-0.1141643
H	-2.7310306	-2.3367054	-0.2340473
O	-1.3321351	-1.0355984	-0.2019331
O	-1.2779456	1.2161294	-0.0727404
H	-0.2804385	1.0401481	-0.1494519

5. B3LYP/D3BJ, planar S_0: (C_{2h} symmetry group):

C	4.3161941	-0.1704406	0.0000000
C	3.2266512	-1.0835242	0.0000000
C	3.4874353	-2.4642783	0.0000000
C	4.7720706	-2.9607877	0.0000000
C	5.8426713	-2.0599784	0.0000000
C	5.6229446	-0.7012644	0.0000000
C	1.8409920	-0.6123149	0.0000000
H	2.6436231	-3.1373073	0.0000000
H	4.9495144	-4.0265536	0.0000000
H	6.8598022	-2.4304154	0.0000000
H	6.4601711	-0.0143523	0.0000000
N	4.1415232	1.1744225	0.0000000
H	4.9374589	1.7827833	0.0000000
H	3.2068204	1.5467148	0.0000000
O	1.5300544	0.5889195	0.0000000
O	0.9235085	-1.5624525	0.0000000
H	0.0073105	-1.1626220	0.0000000
C	-4.3161941	0.1704406	0.0000000
C	-3.2266512	1.0835242	0.0000000
C	-3.4874353	2.4642783	0.0000000
C	-4.7720706	2.9607877	0.0000000
C	-5.8426713	2.0599784	0.0000000
C	-5.6229446	0.7012644	0.0000000
C	-1.8409920	0.6123149	0.0000000
H	-2.6436231	3.1373073	0.0000000
H	-4.9495144	4.0265536	0.0000000
H	-6.8598022	2.4304154	0.0000000
H	-6.4601711	0.0143523	0.0000000
N	-4.1415232	-1.1744225	0.0000000
H	-4.9374589	-1.7827833	0.0000000
H	-3.2068204	-1.5467148	0.0000000

O	-1.5300544	-0.5889195	0.0000000
O	-0.9235085	1.5624525	0.0000000
H	-0.0073105	1.1626220	0.0000000

6. B3LYP/D3BJ, non-planar S_0 (C_i symmetry group)

C	4.2073167	0.9779238	0.0556436
C	3.3986458	-0.1896354	0.0039309
C	4.0154620	-1.4517188	-0.0321649
C	5.3856598	-1.5909909	-0.0128836
C	6.1793371	-0.4403579	0.0454497
C	5.6073380	0.8113569	0.0785697
C	1.9370936	-0.1025547	0.0003074
H	3.3801236	-2.3233587	-0.0726595
H	5.8388373	-2.5714617	-0.0401362
H	7.2580751	-0.5288301	0.0641941
H	6.2326445	1.6946810	0.1187150
N	3.6835060	2.2313442	0.0611331
H	4.2858136	3.0147662	0.2297665
H	2.6855246	2.3353870	0.1415092
O	1.3186509	0.9713571	0.0578239
O	1.3049591	-1.2603163	-0.0649266
H	0.3155006	-1.1182164	-0.0591057
C	-4.2073167	-0.9779238	-0.0556436
C	-3.3986458	0.1896354	-0.0039309
C	-4.0154620	1.4517188	0.0321649
C	-5.3856598	1.5909909	0.0128836
C	-6.1793371	0.4403579	-0.0454497
C	-5.6073380	-0.8113569	-0.0785697
C	-1.9370936	0.1025547	-0.0003074
H	-3.3801236	2.3233587	0.0726595
H	-5.8388373	2.5714617	0.0401362
H	-7.2580751	0.5288301	-0.0641941
H	-6.2326445	-1.6946810	-0.1187150
N	-3.6835060	-2.2313442	-0.0611331
H	-4.2858136	-3.0147662	-0.2297665
H	-2.6855246	-2.3353870	-0.1415092
O	-1.3186509	-0.9713571	-0.0578239
O	-1.3049591	1.2603163	0.0649266
H	-0.3155006	1.1182164	0.0591057

7. B3LYP/D3BJ, planar S_1 (C_s symmetry group):

C	4.3014510	-0.5674150	0.0000000
C	3.1020970	-1.3712140	0.0000000
C	3.2956050	-2.7497400	0.0000000
C	4.5990730	-3.3291170	0.0000000
C	5.7349000	-2.5499810	0.0000000
C	5.5724610	-1.1410160	0.0000000
C	1.7766350	-0.7784040	0.0000000
H	2.4307390	-3.3942320	0.0000000
H	4.6806980	-4.4077850	0.0000000
H	6.7208750	-2.9884810	0.0000000
H	6.4361790	-0.4880770	0.0000000
N	4.1409100	0.7784950	0.0000000
H	4.9356460	1.3952190	0.0000000
H	3.1700730	1.1181880	0.0000000
O	1.5404530	0.4738040	0.0000000

O	0.7774090	-1.6656150	0.0000000
H	-0.0978960	-1.1945140	0.0000000
C	-4.3007290	0.5526360	0.0000000
C	-3.1269460	1.3543590	0.0000000
C	-3.2527910	2.7526640	0.0000000
C	-4.4837780	3.3728040	0.0000000
C	-5.6369150	2.5815300	0.0000000
C	-5.5496250	1.2072490	0.0000000
C	-1.7908170	0.7488070	0.0000000
H	-2.3468320	3.3394310	0.0000000
H	-4.5560990	4.4509490	0.0000000
H	-6.6132290	3.0491310	0.0000000
H	-6.4495020	0.6045700	0.0000000
N	-4.2549950	-0.8032910	0.0000000
H	-5.1048130	-1.3335500	0.0000000
H	-3.3572490	-1.2590450	0.0000000
O	-1.6034720	-0.4779680	0.0000000
O	-0.7874640	1.6028020	0.0000000
H	0.0979490	1.1168090	0.0000000

8. B3LYP/D3BJ, non-planar S_1 (C_1 symmetry group)

C	4.2292206	0.9610156	0.0251375
C	3.3843289	-0.2093377	-0.0145521
C	4.0453655	-1.4339062	-0.0525857
C	5.4691733	-1.5237704	-0.0525668
C	6.2630432	-0.3987340	-0.0146283
C	5.6205993	0.8652255	0.0249178
C	1.9352879	-0.1146582	-0.0146652
H	3.4587549	-2.3386320	-0.0827497
H	5.9208199	-2.5062963	-0.0833288
H	7.3400540	-0.4667238	-0.0146025
H	6.2034620	1.7772640	0.0556703
N	3.6109318	2.1667374	0.0629781
H	4.1418960	3.0207234	0.0922887
H	2.5826570	2.1486797	0.0606371
O	1.2784376	0.9767005	0.0199661
O	1.3070103	-1.2933419	-0.0548838
H	0.3226348	-1.1560156	-0.0516310
C	-4.2269641	-0.9734656	-0.0415525
C	-3.4037821	0.1845123	-0.0180849
C	-4.0059014	1.4529523	-0.0141410
C	-5.3754184	1.6084328	-0.0397026
C	-6.1828787	0.4672217	-0.0718109
C	-5.6244622	-0.7919201	-0.0723295
C	-1.9397280	0.0800319	-0.0138382
H	-3.3595480	2.3172527	0.0056968
H	-5.8164642	2.5949613	-0.0375350
H	-7.2605242	0.5677582	-0.0954897
H	-6.2597050	-1.6689936	-0.0911794
N	-3.7155659	-2.2332915	-0.0092395
H	-4.3230272	-3.0111741	-0.1857560
H	-2.7182399	-2.3424658	-0.1008261
O	-1.3382466	-1.0043845	-0.0516439
O	-1.2956911	1.2286452	0.0277430
H	-0.2965141	1.0804131	0.0227807

9. CAM-B3LYP/D3BJ, S_0 (C_{2h} symmetry group):

C	4.2960895	-0.1696733	0.0000000
C	3.2149871	-1.0789913	0.0000000
C	3.4728973	-2.4547678	0.0000000
C	4.7516254	-2.9501667	0.0000000
C	5.8173858	-2.0510408	0.0000000
C	5.5987284	-0.6981573	0.0000000
C	1.8297379	-0.6113266	0.0000000
H	2.6282719	-3.1261288	0.0000000
H	4.9292422	-4.0150458	0.0000000
H	6.8340719	-2.4206757	0.0000000
H	6.4350741	-0.0114461	0.0000000
N	4.1231239	1.1723078	0.0000000
H	4.9199179	1.7778455	0.0000000
H	3.1907981	1.5469912	0.0000000
O	1.5183829	0.5834939	0.0000000
O	0.9187941	-1.5559395	0.0000000
H	0.0013136	-1.1607752	0.0000000
C	-4.2960895	0.1696733	0.0000000
C	-3.2149871	1.0789913	0.0000000
C	-3.4728973	2.4547678	0.0000000
C	-4.7516254	2.9501667	0.0000000
C	-5.8173858	2.0510408	0.0000000
C	-5.5987284	0.6981573	0.0000000
C	-1.8297379	0.6113266	0.0000000
H	-2.6282719	3.1261288	0.0000000
H	-4.9292422	4.0150458	0.0000000
H	-6.8340719	2.4206757	0.0000000
H	-6.4350741	0.0114461	0.0000000
N	-4.1231239	-1.1723078	0.0000000
H	-4.9199179	-1.7778455	0.0000000
H	-3.1907981	-1.5469912	0.0000000
O	-1.5183829	-0.5834939	0.0000000
O	-0.9187941	1.5559395	0.0000000
H	-0.0013136	1.1607752	0.0000000

10. CAM-B3LYP/D3BJ, S_1 (C_s symmetry group):

C	4.2846063	-0.5583633	0.0000000
C	3.0815520	-1.3709318	0.0000000
C	3.2799169	-2.7494172	0.0000000
C	4.5755979	-3.3102722	0.0000000
C	5.7175391	-2.5263622	0.0000000
C	5.5552259	-1.1290276	0.0000000
C	1.7693655	-0.7866725	0.0000000
H	2.4188011	-3.3977008	0.0000000
H	4.6666079	-4.3876518	0.0000000
H	6.7007597	-2.9683315	0.0000000
H	6.4149321	-0.4718890	0.0000000
N	4.1162101	0.7705317	0.0000000
H	4.9072061	1.3909038	0.0000000
H	3.1457420	1.1070077	0.0000000
O	1.5313765	0.4566628	0.0000000
O	0.7738197	-1.6633813	0.0000000
H	-0.1012720	-1.1939928	0.0000000
C	-4.2814359	0.5518578	0.0000000
C	-3.1159002	1.3497647	0.0000000

C	-3.2376048	2.7430074	0.0000000
C	-4.4620201	3.3627749	0.0000000
C	-5.6108614	2.5736419	0.0000000
C	-5.5257687	1.2052859	0.0000000
C	-1.7804737	0.7463524	0.0000000
H	-2.3303037	3.3269755	0.0000000
H	-4.5335721	4.4400975	0.0000000
H	-6.5863490	3.0413924	0.0000000
H	-6.4254644	0.6038028	0.0000000
N	-4.2389899	-0.8009892	0.0000000
H	-5.0901828	-1.3270691	0.0000000
H	-3.3449366	-1.2608026	0.0000000
O	-1.5928134	-0.4735660	0.0000000
O	-0.7829969	1.5950477	0.0000000
H	0.1016866	1.1113138	0.0000000
